# Microglia recapitulate a hematopoietic master regulator network in the aging human frontal cortex

**DOI:** 10.1016/j.neurobiolaging.2015.04.008

**Published:** 2015-08

**Authors:** Claudia C. Wehrspaun, Wilfried Haerty, Chris P. Ponting

**Affiliations:** a Department of Physiology, Anatomy and Genetics, University of Oxford, Oxford, UK; b Section on Neuropathology, Clinical Brain Disorders Branch, Genes, Cognition and Psychosis Program, IRP, NIMH, NIH, Bethesda, MD, USA; c Department of Physiology, Anatomy and Genetics, MRC Functional Genomics Unit, University of Oxford, UK

**Keywords:** Microglia, Hematopoiesis, Aging, Immune system, Transcriptional network, Master regulator

## Abstract

Microglia form the immune system of the brain. Previous studies in cell cultures and animal models suggest altered activation states and cellular senescence in the aged brain. Instead, we analyzed 3 transcriptome data sets from the postmortem frontal cortex of 381 control individuals to show that microglia gene markers assemble into a transcriptional module in a gene coexpression network. These markers predominantly represented M1 and M1/M2b activation phenotypes. Expression of genes in this module generally declines over the adult life span. This decrease was more pronounced in microglia surface receptors for microglia and/or neuron crosstalk than in markers for activation state phenotypes. In addition to these receptors for exogenous signals, microglia are controlled by brain-expressed regulatory factors. We identified a subnetwork of transcription factors, including *RUNX1*, *IRF8*, *PU.1,* and *TAL1*, which are master regulators (MRs) for the age-dependent microglia module. The causal contributions of these MRs on the microglia module were verified using publicly available ChIP-Seq data. Interactions of these key MRs were preserved in a protein-protein interaction network. Importantly, these MRs appear to be essential for regulating microglia homeostasis in the adult human frontal cortex in addition to their crucial roles in hematopoiesis and myeloid cell-fate decisions during embryogenesis.

## 1 Introduction

The human brain's transcriptome varies over its life span and drastically changes at specific developmental stages, such as at the time of birth and by the end of infancy or childhood (Colantuoni et al., 2011; Kang et al., 2011) or in response to insults or injuries (Hashimoto-Torii et al., 2011; Rosell et al., 2011; VanGilder et al., 2012). In any cell type transcription rate variation allows adaptation to changing environments (Li et al., 2013; Somel et al., 2013) and therefore plays a crucial role in healthy aging or pathogenesis (Courtney et al., 2010; Miller et al., 2010; Mills and Janitz, 2012; Ponomarev et al., 2012; Twine et al., 2011; Vidal et al., 2011). Different cell types and functionally related genes co-ordinate their transcriptional patterns, reflecting the regulatory crosstalk among interacting genes (Konopka et al., 2012; Langfelder and Horvath, 2008; Oldham et al., 2008). A previous network analysis of the human brain's transcriptome from fetal to old age detected transcriptional modules of co-expressed genes that are distinguished mostly by strong fold changes in expression before and after birth and during early childhood (Kang et al., 2011). However, it is less clear how age affects gene modules during the more subtle gene expression changes in later life (Colantuoni et al., 2011; Kang et al., 2011). Identification of age-dependent gene modules in adulthood could improve our understanding of periods that are critical for brain plasticity or for susceptibility to late-onset diseases.

Fine tuning of expression dynamics depends on key regulatory elements for transcription initiation. Transcription factors (TFs) recognize binding motifs in the vicinity of their target genes and control temporal expression patterns (Birney et al., 2007; Wasserman and Sandelin, 2004). The complete set of genes regulated by a given TF is defined as its “regulon”. TFs are highly diverse in their binding patterns and regulon sizes and consequentially in the extent of their potential downstream influence (Wasserman and Sandelin, 2004). A module that is regulated by a TF acting as a master regulator (MR) shows significant overlap between its genes and the TF's regulon (Carro et al., 2010). The identification of MRs in TF cascades is central to understanding the key components underlying transitions between different physiological states (Carro et al., 2010; Della Gatta et al., 2012; Lefebvre et al., 2010). For example, *PU.1, RUNX1,* and *TAL1* act as MRs for hematopoiesis and microglia genesis during embryonic development (Lichtinger et al., 2012). Transcriptional modules have been identified in the human brain's transcriptome based on their link to Alzheimer's disease (Forabosco et al., 2013; Zhang et al., 2013). However, it remains poorly understood which MRs co-ordinate these modules' expression during healthy aging. Importantly, elucidating regulatory patterns for microglia gene expression allows the identification of developmental stages that are critical to disease susceptibility.

Microglia form the innate immune system of the central nervous system (CNS) (David and Kroner, 2011; Norden and Godbout, 2013). They play a fundamental role in healthy and pathological aging (Paolicelli et al., 2011; Schuitemaker et al., 2012), undergoing major morphological changes with increasing age (Conde and Streit, 2006; Hefendehl et al., 2014; Streit et al., 2004) while maintaining cell numbers (Long et al., 1998; Norden and Godbout, 2013; VanGuilder et al., 2011). Transition between their phenotypical activation states, M1 and M2, has been linked to various disease pathways (Appel et al., 2010; Kumar et al., 2013; Liao et al., 2012). M1 reflects the classically activated stage and might contribute to neurotoxicity (Kumar et al., 2013) induced by interferon gamma and toll-like receptor (TLR) agonists (Saijo and Glass, 2011), as opposed to a continuum of alternative activated M2 states involved in tissue repair (Lampron et al., 2011). M2a cells are induced by *IL-4* or *IL-13*, M2b are induced by immune complexes and *TLR* agonists, and M2c are induced by *IL-10* or transforming growth factor (Appel et al., 2010). Animal studies suggest that during aging M1 phenotypes increase, accompanied by a decrease in M2 (Lee et al., 2013). Microglia are key factors for communication between the CNS and the peripheral immune system (Norden and Godbout, 2013), by crosstalking to T-cells (Appel et al., 2010) and by modulating the permeability of the blood-brain barrier (Nishioku et al., 2010; Sumi et al., 2010). During early development, microglia emerge from hematopoietic progenitors in the yolk sac (Ginhoux et al., 2010). Injuries may induce a second wave of hematopoiesis during adulthood and after infiltration of CNS by bone marrow–derived myeloid cells (Lampron et al., 2011; Ponomarev et al., 2013). Microglia from both origins share a dependence on critical TFs, such as *PU.1 (SPI1)* (Ponomarev et al., 2013) which is expressed in the human adult brain (Smith et al., 2013). The microglia response to insults is reflected by dynamic transitions between M1 and M2 phenotypes (Aguzzi et al., 2013) which are mediated via intrinsic (e.g., RUNX1, IRF8, and PU.1) and extrinsic factors interacting with microglia receptors (e.g., CD200, CX_3_CR1, and TREM2) (Kierdorf and Prinz, 2013).

We sought to identify TFs that act as MRs for age-dependent modules within the context of the healthy human postmortem brain. The intact brain environment, including all cell types, is essential for understanding the microglia regulatory network during aging as it evolves in close communication with other cells, for example, neurons (Kierdorf and Prinz, 2013) and the peripheral immune system (Norden and Godbout, 2013). To detect these key regulatory networks in the aging brain, we applied an unbiased data driven approach which first detected modules whose representative expression pattern is correlated with aging and then predicted TFs acting as MRs for these modules. Combining 2 approaches for network analysis, we identified a microglia module whose expression pattern was negatively correlated with age, especially for those genes that encode microglia surface receptors for neuron and/or microglia crosstalk. This microglia module in the healthy adult brain was found to be regulated by a set of hematopoietic MRs, including TFs known to act as endogenous factors regulating microglia activation or cell differentiation during embryonic development (Kierdorf and Prinz, 2013; Prinz and Priller, 2014), such as *RUNX1*, *IRF8*, *PU.1,* and *TAL1*. These MRs, thus, play key roles not only during early development but also in microglia cell homeostasis in the human adult brain.

## 2 Methods

### 2.1 Microarray gene expression

Microarray gene expression data from healthy human controls from 3 independent data sets were used to generate transcriptional networks. In all cases, we averaged multiple probes available for the same annotated gene.

#### 2.1.1 Braincloud

All microarray expression data from the prefrontal cortex were quantified per gene (log_2_[sample/reference]) as relative gene expression and processed as described previously (Colantuoni et al., 2011). The RNA was analyzed using 2-color custom-spotted oligonucleotide microarrays from the National Human Genome Research Institute microarray core facility using the Illumina Oligoset (HEEBO7) of 49,152 70-mer probes (Colantuoni et al., 2011). We included n = 184 subjects, aged 12.9–78.2 years. If multiple probes were available for 1 gene, we calculated the average expression for this gene, resulting in a total of n = 18,846 available genes (GSE30272). The representative expression patterns of the transcriptional modules were tested for correlation with the available confounding variables RNA integrity number (RIN), pH, and postmortem interval (PMI).

#### 2.1.2 Medical Research Council sudden death brain and tissue bank

If multiple probes were available for 1 gene, we calculated the average expression for this gene, resulting in a total of n = 21,513 probes from frontal cortex of n = 97 subjects, aged between 16 to 83 years (available from GSE46706 using Affymetrix Human Exon 1.0 ST Array) (Millar et al., 2007, Trabzuni et al., 2011). Background correction and array preprocessing were performed using multiarray average quantile normalization. Quantile normalization is a part of the robust multiarray average expression summary measure and aims to normalize a batch of arrays to allow for meaningful comparisons. The representative expression patterns of the transcriptional modules were tested for correlation with the only available confounding variable, PMI.

#### 2.1.3 Harvard brain tissue resource center

Gene expression data from the dorsolateral prefrontal cortex of n = 100 healthy subjects between 50 and 95 years were included (GSE44770 using Rosetta/Merck Human 44k 1.1 microarray) (Zhang et al., 2013). N = 14,400 probes with annotated gene names were used of n = 30,305 available probes. Expression was quantified as log_2_(sample/reference) as relative gene expression.

### 2.2 ChIP-Seq data

We used publicly available ChIP-Seq data to validate TF targets. If available, we annotated peaks from cell lines provided by ENCODE (Birney et al., 2007); alternatively we searched for ChIP-Seq data for MRs described in the literature. The publicly available data provided ChIP-Seq peak sets. All peaks were annotated using HOMER [v4.3 (Heinz et al., 2010)]. (1) For PU.1 (SPI1), we used broad ChIP-Seq peaks from 3 cell lines in the ENCODE data: GM12878 (B-lymphocyte, lymphoblastoid; 42,935 peaks), GM12891 (B-lymphocyte, lymphoblastoid; 48,830 peaks), and K562 (leukemia; 28,677 peaks). (2) RUNX1 peaks were annotated from peripheral blood mononuclear cells CD34+ and transferred from build hg18 to hg19 using the online liftOver tool [PBMNC CD34+; sample GSM722708 (Ptasinska et al., 2012); 14,536 peaks], and from cell line CUTLL1 (T lymphoblastic leukemia and/or lymphoma cell line; sample GSM1252931; 28,156 peaks) (3) IRF8 ChiP-Seq peaks were retrieved from human germinal center B cells (LY1 cell lines). We included only peaks in IRF8-target genes which were replicated across all cell lines used in the original study (183 peaks) (Shin et al., 2011). Peaks were transferred from build hg18 to hg19 using the online liftOver tool (part of the UCSC Genome Browser). (4) ChiP-Seq peaks from K562 cell ENCODE data from 2 replicates were used for TAL1 (26,255 and 54,782 narrow peaks). In addition, we used genomic regions occupied by TAL1 in 4 other cell lines: Jurkat, CCRF-CEM (human T cell lymphoblast-like cell line), and PrimaGRAFT (Prima 2 and Prima 5, derived from primary T-ALL cells in immunocompromised mice and mapped to hg18) (Sanda et al., 2012) (19,844 occupied regions). The co-ordinates of the occupied regions were transferred from genome-build hg18 to hg19 using the online liftOver tool. (5) For NANOG, we used narrow peaks (n = 5473) from cell line H1-hESC (human embryonic stem cells) from the ENCODE data.

### 2.3 Weighted correlation network analysis

Coexpression networks were built using step-by-step network construction and module detection as implemented in WGCNA [Weighted correlation network analysis (Langfelder and Horvath, 2008)]. Briefly, in the first step a pairwise Pearson's correlation matrix of all genes across all time points was calculated. This correlation matrix was then converted into an adjacency matrix. The soft thresholding power was chosen to reach a scale-free topology fit index indicating approximate scale-free topology, as suggested by the authors (Langfelder and Horvath, 2008). Next, the adjacency matrix was transformed to a topological overlap matrix, thereby reducing spurious associations, and a dissimilarity matrix was calculated, which was then used to produce a hierarchical tree. The minimum cluster size was arbitrarily set to 10 genes. The standard method for branch cutting, namely Dynamic Tree Cut, was applied (Langfelder and Horvath, 2008). This function returns gene modules, each of which is assigned a color. To reduce redundant information, modules with highly similar expression profiles were merged. This merging procedure was applied using a height cut of 0.25 (corresponding to a correlation of 0.75). Merging was based on the comparison of the module eigengenes which provides a summary expression metric. These module eigengenes were also correlated with any external quantitative trait to analyze the dependency between the given trait and the module's expression. Here, we used age and 3 potential confounding variables (RIN, pH, and PMI). More specifically, we selected modules whose eigengene was correlated with age at more significant *p*-values than for available confounding variables. For each available confounding variable, we further tested for Pearson's correlation between age and module eigengene after matching the samples for the available confounding variables. The matching procedure consisted in first selecting samples with the same value for a confounding variable, and subsequently testing for correlation of the module eigengene using these samples only. The transcriptional networks were undirected, or in other words did not contain information on edge orientation. Similar results were found, however, for directed networks (data not shown).

### 2.4 ARACNE

Algorithm for the Reconstruction of Accurate Cellular Networks (ARACNE) uses mutual information (MI) to reconstruct transcriptional networks and permits the prediction of TF targets (Basso et al., 2005; Margolin et al., 2006a, 2006b). Reverse engineering yields a scale-free hierarchical gene-gene coexpression network (Basso et al., 2005). Briefly, “scale-free” refers to a network with a small number of major hub genes. Importantly, ARACNE takes an unbiased approach which uses the full dynamic range of the data without assuming the underlying network topology (Margolin et al., 2006b). The construction of a network from genome-wide expression data is based on the definition of statistical dependencies between all variables, such as expression time courses. ARACNE estimates MI using a Gaussian kernel as described further in (Margolin et al., 2006a). Redundant network edges are pruned using data processing inequality (DPI). DPI tests each gene triplet, irrespectively of whether edges in this triplet have been examined before, and removes edges with the lowest MI. How many triplets are broken depends on the chosen DPI tolerance, ranging from 0, where all triplets are broken on the weakest link, to 1 which preserves all triplets; recommended values are between 0 and 0.15 (Margolin et al., 2006b); here we applied 0.1. If a list of known TFs is provided as a prior, putative TF-target connections are protected in such a way that edges between a given TF and its target cannot be eliminated in favor of edges between 2 nonTFs (Margolin et al., 2006b). We provided a list of n = 2001 TFs previously described in Vaquerizas et al. (2009) and Wingender et al. (2013). ARACNE converts a user-provided *p*-value to an MI threshold [*p*-value = 10^−7^ (Margolin et al., 2006a, 2006b)]. Subsequently, ARACNE reports only MI values that are more significant than this threshold, which represents a probability of 10^−7^ to obtain an MI score greater or equal to the calculated threshold by chance if the expression levels of 2 genes vary independently of each other (Margolin et al., 2006b). As suggested by the authors (Margolin et al., 2006b), we generated a consensus bootstrapping network using n = 100 sampling steps.

### 2.5 MR analysis

To identify TFs acting as MRs, we predicted the regulon (in other words the target genes) of each TF using ARACNE. Next, we used Fisher's exact test (FET) to calculate whether the intersection of the age-dependent coexpression module with each TF's regulon was larger than expected by chance (α < 0.05, corrected in each data set for the number of TFs that were included in the transcriptional network constructed by ARACNE). More precisely, the contingency table for the FET contained genes in the module (yes and/or no) versus genes in the TF's regulon (yes and/or no). TFs whose regulon shared a higher number of genes with the age-dependent module than expected by chance were termed “master regulators” for the age-dependent module.

### 2.6 Enrichment of functional categories and cell types

Enrichment was assessed using Gene Ontology (GO)–terms and Kyoto Encyclopedia of Genes and Genomes (KEGG)-pathways comparing the genes in the microglia module against a background of all genes in the WGCNA network using the functional classification tool Database for Annotation, Visualization, and Integrated Discovery (Huang et al., 2009) and Genomic Regions Enrichment of Annotations Tool [GREAT version 2.0.2 (McLean et al., 2010)]. Briefly, GREAT assigns “basal regulatory domains” as defined by McLean et al. (2010) to the genomic regions of interest, spanning 5 kb upstream to 1 kb downstream of the transcription start site (TSS) of the nearest gene relative to the regions provided in the input, plus a distal extension of up to 1000 kb or up to the next nearest gene's basal regulatory domain in both orientations. We included GO-terms from the analysis using GREAT with a significant q-value (α < 0.05, hypergeometric *p*-value, corrected for multiple testing using false discovery rate [FDR]) and KEGG-pathways in Database for Annotation, Visualization, and Integrated Discovery with a significant *p*-value (α < 0.05, Bonferroni corrected). The genes contributing to the age-dependent module for all 3 data sets combined (n = 407 basic regulatory domains available in ENSEMBL BioMart) were compared with a background of all genes with annotated gene names and basal regulatory regions in all 3 data sets (n = 19,045). Similar results were found when enrichment was tested separately for each of the 3 data sets. The underlying cell population of the age-dependent gene coexpression module was tested by comparing the number of expressed microglial markers in the module combined for all 3 data sets to the number of expressed microglial markers in n = 1000 random gene sets of the same sample set size using the genes available in all 3 microarrays combined as a background. For testing enrichment of the named ontologies for MRs (n = 107, united for all 3 data sets), we used all genes with annotated gene names and basal regulatory regions in all 3 data sets as a background (n = 19,045 genomic regions).

### 2.7 Disease Association Protein-Protein Link Evaluator

DAPPLE (Disease Association Protein-Protein Link Evaluator) takes as input a list of genes (here 107 MRs) and searches for physical connectivity between proteins based on protein-protein interactions known from the literature. DAPPLE assesses *p*-values for direct and indirect interactions using n = 1000 within-degree node-label permutations (Rossin et al., 2011). “Degree” of a node refers to the number of edges directly connected to the node. An empirical *p*-value is calculated for each node as the number of random permutations which yield a degree for the node which is as high as or higher than the observed node degree, divided by the total number of permutations (Rossin et al., 2011).

### 2.8 Intersection of genomic intervals

We used the GAT (Genomic Association Tester) tool (Heger et al., 2013) to test for a significant intersection between the basal regulatory regions (5 kb upstream to 1 kb downstream of the TSS) of the genes in the age-dependent module and the ChIP-Seq peaks for the TFs that were identified as MRs for the age-dependent module. GAT calculates the intersection of 2 sets of genomic intervals while controlling for different potential confounders such as varying G + C content or differences among chromosomes (Heger et al., 2013). It assigns a *p*-value based on a randomization simulation procedure within the provided workspace–potentially the whole genome, or a pruned background. Here, we used the basal regulatory regions of all 3 gene expression data sets that were used to build the transcriptional networks. More precisely, GAT takes the 2 sets of interest, for example, ChIP-Seq peaks and the co-ordinates for genes of interest, and compares their overlap to randomly chosen sets of intervals across the workspace. Multiple testing correction was applied using Storey's q-value (Storey and Tibshirani, 2003) or Benjamini-Hochberg correction (Benjamini et al., 2001) by an false discovery rate procedure (Heger et al., 2013).

## 3 Results

### 3.1 An age-dependent gene module in frontal cortex is preserved across independent data sets

**Fig. 1:**
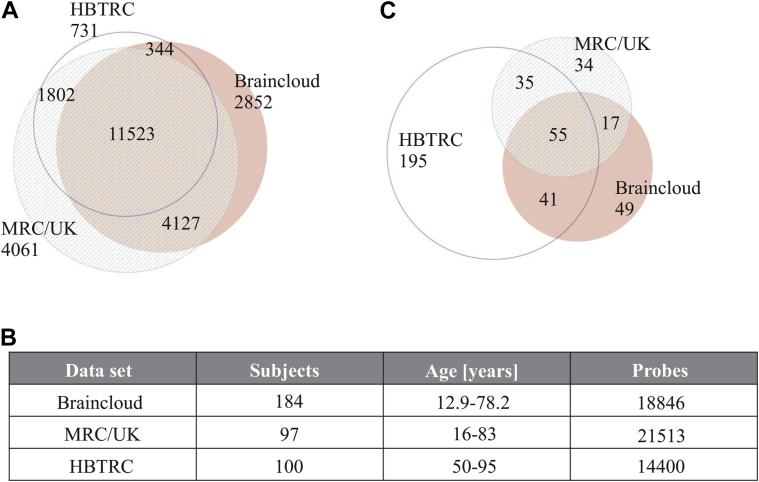
Venn diagram showing the overlap of genes in the 3 microarray gene expression data sets and in the age-dependent module. (A) Overlap of genes from the 3 microarray gene expression data sets braincloud, Medical Research Council (MRC/UK) and Harvard Brain Tissue Resource Center (HBTRC). (B) Demographics and coverage of the 3 data sets. (C) Venn diagram showing the age-dependent modules and their gene overlaps.

Three independent microarray gene expression data sets from healthy human postmortem frontal cortex tissue (Fig. 1A–B) were used to generate transcriptional networks across the life span using WGCNA (Langfelder and Horvath, 2008). Genes whose expression is distinctive of cell types and/or are functionally related are expected to show higher co-ordination of expression and thus can be assembled by this analysis into gene modules (Langfelder and Horvath, 2008). Age-dependent modules were detected using an unbiased approach, focusing only on modules whose representative expression pattern, the module's eigengene (Langfelder and Horvath, 2008), was significantly correlated with age (α < 0.05) at higher significance levels than potentially confounding variables, namely RIN, pH, and PMI. Only 1 module fulfilled this criterion in all 3 data sets (Supplementary Tables 1–3) and showed strong enrichment for gene annotations linked to the immune system in the GO data base (Supplementary Table 4) for all the 3 data sets used (Fig. 1C).

**Fig. 2:**
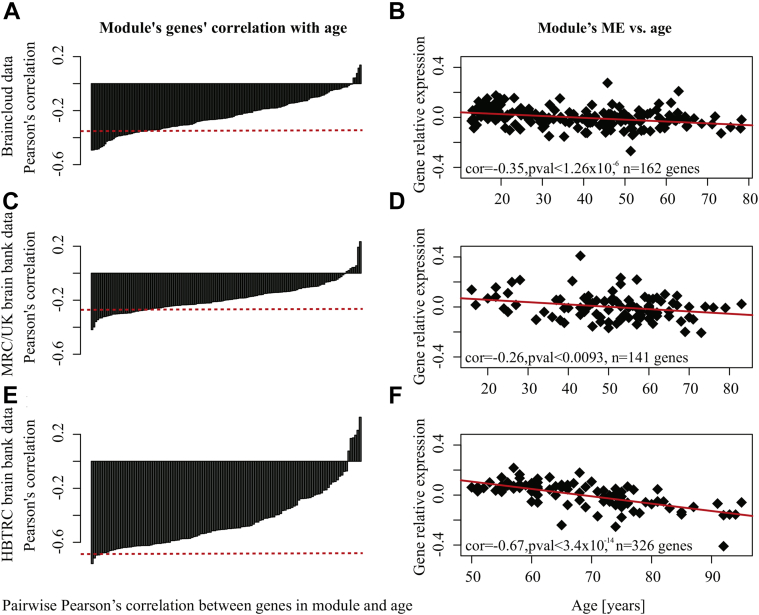
The age-dependent modules' eigengene's [gene relative expression: log_2_(sample/reference)] negative correlation with age. (A) Bar plot for Pearson's correlation (y-axis) of the expression of all n = 162 genes (x-axis) in the age-dependent module with age, derived from braincloud data ranked by increasing correlations. Each bar reflects the Pearson's correlation of a given gene's expression level with age. The red dashed line indicates the Pearson's correlation for the age-dependent module's eigengene with age (Pearson's correlation = −0.35). (B) A negative slope of a linear model fit with the age-dependent module's eigengene's expression as the dependent variable, and age [years] as the independent variable. (C) and (D) display the same metrics for the network derived from MRC/UK data (n = 141 genes in the age-dependent module); (E) and (F) correspondingly display the same metrics for the network derived from HBTRC data (n = 326 genes in the age-dependent module). Abbreviations: HBTRC, Harvard Brain Tissue Resource Center; MRC, Medical Research Council.

The module's eigengene was negatively correlated with age at lower *p*-values than with confounding variables in each data set (Fig. 2 and Supplementary Fig. 1).

We validated the negative correlation between module eigengene and age after matching samples for the confounding variables. “Matched” in this context refers to the testing for correlation between the module eigengene and age just for those samples that share the same value for the potentially confounding variable. Supplementary Fig. 1 illustrates an example of Pearson's correlation coefficients between module eigengene and age (in years) for constant RIN values in the braincloud data. Clearly, for most of the RIN values the negative correlation between module eigengene and age (Fig. 2) is replicated (Supplementary Fig. 1). Similar results were found for pH-values and when Spearman's correlation was calculated instead of Pearson's correlation. For simplicity, the following analysis is described for the age-dependent module combined across all 3 data sets. The “combined module” included n = 426 genes that were expressed in at least 1 of the 3 data sets (Fig. 1C).

### 3.2 Microglia surface receptors' expression levels reflect the age-dependence of the module

To determine whether a specific cell type can explain this age-dependent module, through variation of either its population size or its gene expression repertoire, we sought known gene markers that specify different CNS cell types (Butovsky et al., 2014; Cahoy et al., 2008; David and Kroner, 2011; Forabosco et al., 2013) (Supplementary Table 5). The module displayed a highly significant 20-fold enrichment for microglial markers compared with random gene sets of identical size (40 of 162 microglia markers, compared with a median of 2 expected by chance, empirical *p*-value < 0.001); moreover, it contained only 1 of the n = 29 markers specific for neurons, oligodendrocytes, endothelial cells, or astrocytes. The 3 data sets showed similar numbers of expressed microglia marker genes in the age-dependent module. More specifically, about 10% of genes were microglia markers in the “combined” module as well as in the braincloud (n = 19 expressed microglia markers) and Harvard brain tissue resource center (HBTRC) data sets (n = 28 expressed microglia markers), compared with a slightly higher number of microglia marker genes in the module derived from the Medical Research Council (MRC)/UK data set (about 20% microglia marker genes [n = 27] in the age-dependent module; see Supplementary Tables 1–3 and 5). The module contained 4 of 6 gene markers (*P2RY12*, *GPR34*, *C1QA,* and *PROS1*) that have been validated as being expressed in human adult microglia but not in human blood-derived monocytes (Butovsky et al., 2014). Importantly, the age-dependent module contained a subset of these 4 markers in each data set (*P2RY12*, *GPR34*, and *C1QA* in the MRC/UK data set, *C1QA* and *PROS1* in the HBTRC data set, and *GPR34* and *C1QA* in the braincloud data set). This “microglia module” thus principally reflects gene expression variation across the human frontal cortex life course in microglia, and not in other CNS cell types, and is not derived from blood contamination.

**Fig. 3:**
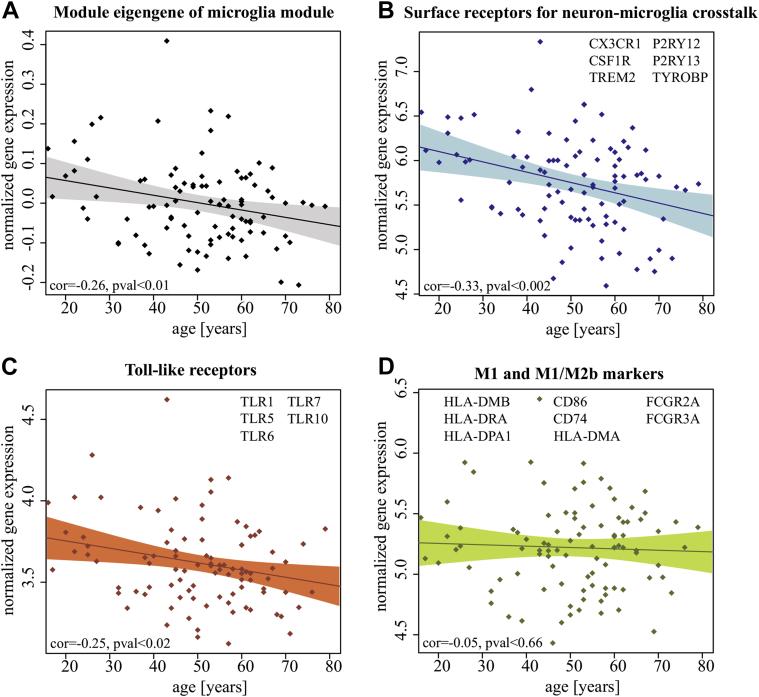
Microglia surface receptors' and microglia M1 and M1/M2b phenotype markers' expression correlation with age for Medical Research Council/United Kingdom (MRC/UK) data. The regression model fit and confidence interval for normalized gene expression versus age is for (A) the microglia module's eigengene (shown in gray), (B) surface receptors for neuron-microglia crosstalk (blue), (C) toll-like receptors (red), and (D) M1 and M1/M2b activational phenotype markers (green). The x-axis shows age and the y-axis shows normalized gene expression of the microglia module's eigengene. Legends in (B–D) indicate all respective marker genes that are expressed in the MRC/UK data set and therefore were included in the regression. Pearson's correlation coefficient of the mean expression of the expressed marker genes (B–D) or module eigengene versus age (A) is shown in the bottom-left corner of each plot.

We next considered whether the module contains genes that are highly expressed in particular microglial subtypes, more specifically 62 genes that encode known markers for M1 or M2 phenotypes (Supplementary Table 5). The combined microglia module contained marker genes predominantly for M1 or M1/M2b phenotypes. Nevertheless, these markers did not display a negative correlation with aging (Fig. 3; Supplementary Fig. 2). To test whether the observed independence of age was specific to the markers for microglia subtypes that were assigned to the microglia module or true for all available markers, we calculated the mean correlation between all marker genes for a specific microglia subtype and age across the life span for the MRC/UK and the braincloud data (Supplementary Figs. 3 and 4). Only markers specific for the anti-inflammatory phenotype (M2a) showed a significant decrease of gene expression in both data sets across the life span (α < 0.05, uncorrected *p*-values). A decrease in expression of M2a-markers, combined with relatively stable expression of M1 markers, is in line with previous studies indicating a switch from an M2- to M1-skewed microglial activation during aging (Varnum and Ikezu, 2012). However, in contrast to M1 markers, M2a-markers were not assigned to the age-dependent microglia module in both the MRC/UK and the braincloud data, but were distributed across a relatively large number of modules. This indicates that expression of M2a markers decreases during ageing, yet is more stochastic than the expression of genes assembled in the age-dependent microglia module.

Microglia surface receptors for microglia-neuron crosstalk as well as TLRs for communication with pathogen-associated molecular patterns (Kierdorf and Prinz, 2013) tended to show a negative correlation between gene expression level and age (Fig. 3; Supplementary Fig. 2A). The importance of microglia surface receptors for communication between microglia and neurons is, for example, reflected by *CX*_*3*_*CR1*'s nonredundant role for direct contact with synapses and subsequent removal of synapses by phagocytosis (Kierdorf and Prinz, 2013; Paolicelli et al., 2011) or *TREM2*'s effect on autoimmune CNS demyelination (Kierdorf and Prinz, 2013; Takahashi et al., 2007).

Our data are in line with previous observations of decreased expression of the microglia-specific surface receptor *CX*_*3*_*CR1* in the aged brain (Harry, 2013; Wynne et al., 2010). This trend was less pronounced, however, in the data for subjects aged 50 years and older (HBTRC, Supplementary Fig. 2B), and was not observed when only considering subjects 50 years and older in either MRC/UK or braincloud data sets. This indicates that expression of these receptors decreases over the early adult life span but remains relatively constant within the oldest age group. These gene expression time courses indicate that the age-dependency of the microglia module are driven by expression changes of intrinsic factors that regulate the reception of exogenous signals for microglia activation, especially in microglia-neuron crosstalk (Kierdorf and Prinz, 2013; Saijo and Glass, 2011).

### 3.3 A network of MRs controls the microglia module

To detect microglia-specific TFs in the healthy adult human brain, which we shall refer to as MRs (Carro et al., 2010), TF targets were predicted by reconstructing a transcriptional network using ARACNE (Margolin et al., 2006a, 2006b). MRs for the microglia module were identified based on significant overlap of a TF's predicted regulon with the microglia module ([FET], α < 0.05, corrected for 1655 TFs in the ARACNE-derived transcriptional network). Importantly, using this approach, TFs can be identified that are not part of the microglia module themselves, for example, because they function as upstream regulators. N = 42, 23, and 64 MRs were identified in the 3 data sets, resulting in n = 107 MRs in all 3 sets combined (Supplementary Table 6). Five MRs–*TAL1, PLEK, RUNX1, IRF8,* and *ZFP36L2* – were replicated in all 3 data sets. Twelve more were identified in at least 2 data sets (*MAF, FLI1, KLF6, HHEX, LYL1, PARP12, BNC2, PRDM4, IFI16, ELK3, FOS,* and *NFATC2*).

**Fig. 4:**
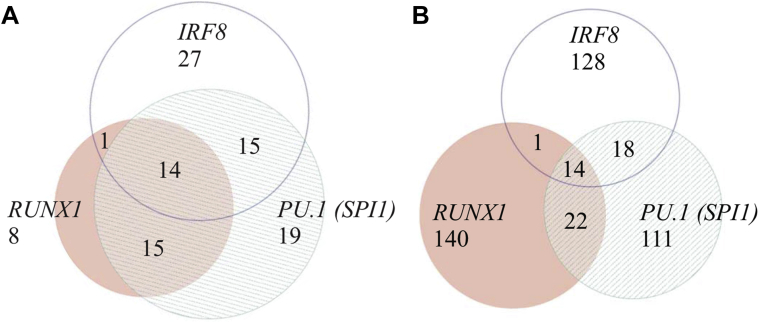
Overlap of MR's (RUNX1, PU.1, and IRF8) predicted target genes in the microglia module (A) compared with genes from the whole data set (Medical Research Council/United Kingdom [MRC/UK]) (B). (A) Predicted regulons (in other words target genes) of *RUNX1, PU.1,* and *IRF8* in the MRC/UK gene expression data. (A) displays only genes that are part of the microglia module. (B) As in (A) but including all predicted target genes of the 3 MRs across all genes in the network. It is of note that n = 14 genes that are regulated by all 3 MRs are present in both (A) and (B). Supplementary Fig. 5 shows the same gene sets as in this figure including all gene names.

The top-ranked MRs include TFs that are known to be expressed in microglia (Kierdorf et al., 2013; Smith et al., 2013) and act in experimentally validated interactions. For example, RUNX1 and PU.1 (that was top-ranked in the MRC/UK data but not replicated) form a negative feedback loop for regulating *PU.1* transcript abundance and subsequently regulate myeloid cell fate (Jin et al., 2012; Kierdorf and Prinz, 2013), and PU.1 and IRF8 function as interaction partners in microglia (Kierdorf et al., 2013). RUNX1 also directly interacts with TAL1 and FLI1 (Lichtinger et al., 2012), whereby PU.1 in turn mediates TAL1 activity (Le Clech et al., 2006). The interdependence among the MRs is further illustrated by the strong overlap of their regulons (Fig. 4 and Supplementary Fig. 5). Almost all predicted target genes that are regulated by at least 2 of the MRs RUNX1, PU.1, and IRF8 belong to the microglia module (Fig. 4). More specifically, all 14 genes that are regulated by all 3 MRs, RUNX1, PU.1 and IRF8, belong to the microglia module (Fig. 4A). This is in contrast to most of the genes that are regulated by only 1 of these 3 regulators which tend not to be members of the microglia module (Fig. 4B). In addition to the trivial GO-enrichment for regulatory functions of TFs, 107 MRs were also significantly enriched in KEGG-pathways “Acute myeloid leukaemia” (*p*-value = 2.5 × 10^−3^, Bonferroni corrected) and “Pathways in cancer” (*p*-value = 6.9 × 10^−3^, Bonferroni corrected) terms. These findings likely reflect their crucial roles in myeloid and hematopoietic cell-fate decisions (Lichtinger et al., 2012).

**Fig. 5:**
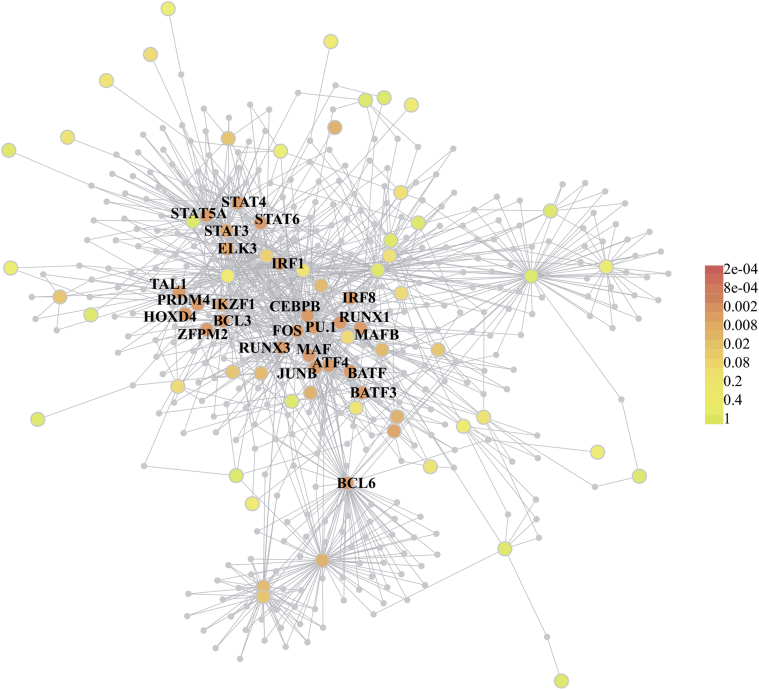
Protein-protein interactions are recapitulated in a MR (MR) network. A protein-protein network, derived from direct and indirect interactions among proteins encoded by MRs' genes. Colors represent Disease Association Protein-Protein Link Evaluator *p*-values (see legend), calculated from 1000 within-degree node-label permutations. The network was constructed on the basis of physical protein-protein interactions that have been reported in the literature (Rossin et al., 2011). For simplicity, only names for proteins with the most significant interactions are displayed; for the complete set see Supplementary Table 7.

Next we asked whether transcriptional level interactions, as derived above via network analysis, are also manifested on the protein level. We tested whether the 107 MRs engage in known protein-protein interactions. For this, we used DAPPLE to search for significant physical connectivity, based on protein-protein interactions that have been reported in the literature (Fig. 5) (Rossin et al., 2011). DAPPLE assesses the significance of interactions in the input gene sets based on node-label permutations, as described in the Section 2 (Rossin et al., 2011). We found that 40 of 107 MRs reached a corrected *p*-value < 0.05 (Supplementary Table 7), including 3 of the 5 MRs that were replicated in every data set (TAL1, RUNX1, IRF8) and the top-ranked MR in the MRC/UK data set (PU.1). Protein-protein network statistics were highly significant, showing a higher number of direct connections between MRs than expected by chance (n = 48 direct interactions between 36 proteins in direct network, *p-*value < 0.001), higher average direct connectivity of MRs (or direct binding degree; mean = 2.7, *p-*value < 0.001), as well as indirect connectivity (mean = 50.7, *p-*value < 0.001). The protein-protein interaction network derived by DAPPLE indicates that interactions between the MRs of the microglia module act not only on the transcriptional but also on the protein level.

### 3.4 ChIP-Seq data indicate causal roles of MRs for the microglia module

**Fig. 6:**
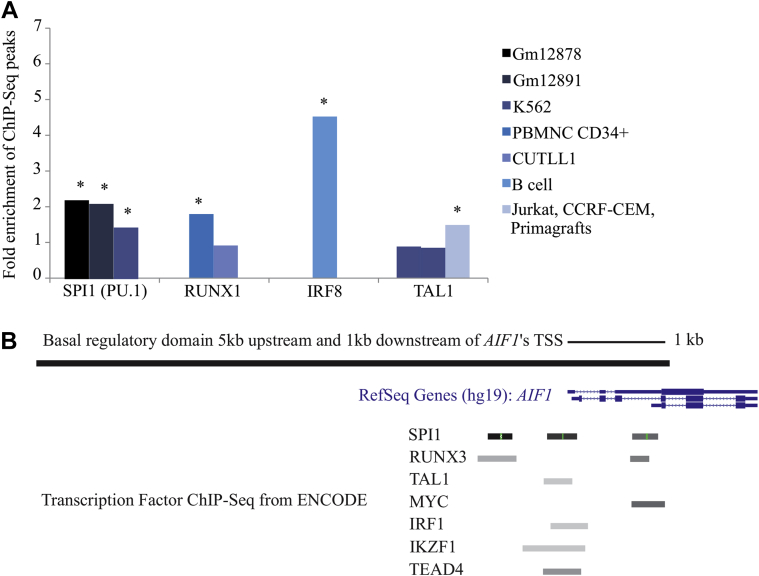
ChIP-Seq peaks for SPI1 (PU.1), RUNX1, IRF8, and TAL1 near to genes in the combined microglia module. (A) Fold enrichment for SPI1 (PU.1), RUNX1, IRF8, and TAL1 ChIP-Seq peaks in all annotated basal regulatory domains of all genes in the combined microglia module (n = 407 assigned to the microglia module in all 3 data sets combined). Asterisks denote a significant (*p-*value < 0.05) enrichment. The legend indicates the cell lines that were used for each MR. (B) Illustration of MR binding sites overlapping the basal regulatory domain of *AIF1* (taken from the UCSC genome browser).

The predicted target genes of the MRs could reflect either indirect (noncausal) co-ordination among genes in the transcriptional network, or direct (causal) interactions of TFs and their target genes. To distinguish between these possibilities, we verified that the MRs' targets in the microglia module predicted by ARACNE were causal using publicly available ChIP-Seq data for RUNX1, IRF8, and TAL1, TFs that were replicated in all 3 data sets, and PU.1, which was top-ranked in the MRC/UK set, but not replicated (Fig. 6). For each of the 4 MRs, we tested for significant overlap between their binding sites as indicated by their ChIP-Seq peaks, and the basal regulatory domains [5kb upstream and 1kb downstream of each gene's TSS (McLean et al., 2010)] of their predicted target genes in the combined microglia module (n = 407 basic regulatory domains available in ENSEMBL BioMart). The overlap between ChIP-Seq peaks and the combined microglia module was compared with a background of 19,045 basal regulatory domains of all genes available in ENSEMBL BioMart and expressed in at least 1 of the 3 microarray gene expression data sets. As a negative control, we tested for overlap between ChIP-Seq peaks of a randomly chosen TF which was not identified as a MR (NANOG) and basal regulatory domains of all expressed genes from the combined microglia module.

PU.1 is known to bind near the promoter regions of its target genes (Pruitt et al., 2005). ChIP-Seq peaks for PU.1 binding annotated within TSS-promoter regions in 3 available cell lines from the ENCODE data (GM12878, GM12891, and K562) (Birney et al., 2007) showed significant overlap with the genes in the combined microglia module (Fig. 6A; all significant *p*-values < 0.002) in each of the 3 cell lines. Enrichment of PU.1 binding sites in the combined microglia module validates PU.1 as a correctly identified MR although it was only detected in 1 data set, and is in line with previous studies (Satoh et al., 2014). Correspondingly, ChIP-Seq peaks for RUNX1, IRF8, and TAL1 indicated significant overlap of these MRs' binding sites with the basal regulatory domains of the genes in the combined microglia module (Fig. 6). In contrast, ChIP-Seq peaks for a randomly chosen TF (NANOG), which was not identified as a MR, did not significantly overlap with the basal regulatory domains of the genes in the combined microglia module (*p-*value > 0.05). For the cell lines which had gene expression data available, namely the cell lines provided by the ENCODE data, analyses were repeated and similar results were found after the brain-expressed genes from the human postmortem data were restricted to the genes whose expression was supported by at least 1 sequencing read in the respective cell line.

The significant overlap of TF binding sites as detected by ChIP-Seq and the combined microglia module indicates a causal role for the identified hematopoietic MRs in the expression of their target genes. As assembly of the microglia module as well as prediction of microglia-specific MRs were founded upon gene expression changes across the human adult life span, the present data show that each of the identified MRs plays a key role in microglia homeostasis not only during embryogenesis but also in the aging brain.

## 4 Discussion

Regulation of microglia homeostasis in the healthy adult human brain is poorly understood (Kierdorf and Prinz, 2013). Previous studies have elucidated how microglia emerge from the yolk sac during embryogenesis, and are dependent on the orchestrated functions of TFs, including RUNX1, PU.1, and IRF8 (Ginhoux et al., 2010; Kierdorf et al., 2013). In response to insult or injury, microglia maintain the ability to self-renew (Ajami et al., 2007) or monocytes may be recruited to differentiate into microglia from the bone marrow (Lampron et al., 2011; Ponomarev et al., 2013). Studies that have mostly been performed in cell lines or animal models provided evidence that key TFs are also necessary for regulating the transition between microglia activational phenotypes, specifically for the transition between proinflammatory (M1) and anti-inflammatory (M2) states (Jin et al., 2012; Kierdorf and Prinz, 2013; Parakalan et al., 2012; Zusso et al., 2012). However, it remains unclear what role these key regulators play in the homeostasis of microglia in the healthy adult human brain (Kierdorf and Prinz, 2013).

Here we have shown that a gene coexpression module, which is highly enriched for microglia markers (predominantly for M1 and M2b phenotypes), is controlled by a network of MRs that also act as key players during hematopoiesis and myeloid cell lineage determination. The microglia module was identified in 3 independent data sets based on the negative correlation of gene expression with aging and contained genes encoding receptors for exogenous signals for microglia activation: CX_3_CR1 transmits “resting” signals, TREM2 and TYROBP initiate phagocytosis, purinergic receptors (P2RY12, P2RY13, P2RY6, P2RY5) signal neuronal injury and CSF1R induces cell survival or proliferation (Kierdorf and Prinz, 2013). More specifically, the expression levels of genes encoding microglia surface receptors for microglia-neuron crosstalk or TLRs were negatively correlated with increasing age. The predicted MRs were not all members of the microglia module, and included subsets of endogenous TFs (e.g., RUNX1, PU.1, and IRF8) that are known to regulate microglia activation (Kierdorf and Prinz, 2013), and are required for differentiation during embryonic development (Kierdorf et al., 2013; Lichtinger et al., 2012). These TFs act co-ordinately with highly overlapping binding sites. For example, an IRF8 and PU.1 heterodimer exhibits stronger binding activity than IRF8 alone (Kubosaki et al., 2010; Shin et al., 2011). Importantly, our analysis demonstrates that interacting hematopoietic MRs from early development are recapitulated within adult human brain tissue. This indicates that a number of the key factors acting during early microglia development—including RUNX1, PU.1 and IRF8—also play crucial roles in adult microglia homeostasis.

Previous work has provided evidence for a cohesive immune-microglia module that is evident in human brain transcriptomes. These studies compared the transcriptomes of different neuroanatomical subdivisions (Forabosco et al., 2013; Hawrylycz et al., 2012; Oldham et al., 2008; Zhang et al., 2013), or searched for candidate genes for susceptibility to neurodegenerative disease (Forabosco et al., 2013; Zhang et al., 2013). For example, *TREM2* is a predisposing factor for late-onset Alzheimer's disease (Forabosco et al., 2013) and was also present in the microglia module in all 3 data sets in the present study. However, although *TREM2* showed highly significant module membership, in other words correlation with the module eigengene, it was not ranked in the top 10% of microglia module genes with highest module membership across the 3 data sets and thus would not have been selected as a central hub gene in an objective, data-driven approach. It has to be noted, however, that we find that it is not a central hub from frontal cortex data, whereas *TREM2* was identified in previous work as a major hub in different brain regions (Forabosco et al., 2013; Jiang et al., 2014) with increased expression in a subset of these regions during aging (Forabosco et al., 2013; Jiang et al., 2014). Furthermore, previous studies investigating various areas in human postmortem brains from 55 subjects aged 20–99 years (hippocampus, entorhinal cortex, superior-frontal gyrus, and postcentral gyrus) reported the upregulation of genes related to inflammation and immune components (Berchtold et al., 2008; Cribbs et al., 2012). Along these lines, despite general consensus that the number of microglia does not increase with age (Norden and Godbout, 2013), single studies reported microglia becoming more numerous in distinct brain regions during normal aging, namely cortical sensory areas (Tremblay et al., 2012). These results could indicate a regional specificity for immune-related transcriptional regulation in the adult human brain. Further studies focused on comparing transcriptomes from various postmortem brain areas, yet these were derived from both healthy controls and disease cases. In one of these studies, *TYROBP* was identified as a key regulator for an immune- and microglia-specific transcriptional network, that was differentially expressed between control and late-onset Alzheimer's disease postmortem brains, highlighting its potential role in disease status (Zhang et al., 2013).

Changes in transcript abundance can be caused by changes in transcription rate in the cell, or by changes in relative or absolute cell numbers of a specific cell type (Ponting and Oliver, 2012). Previous lines of work have shown that—although the relative number of glia cells compared with neurons increases with age (Terry et al., 1987)—the cell number of microglia stays stable during the adult life time (Norden and Godbout, 2013; VanGuilder et al., 2011). Furthermore, as the microglia module predominantly contained markers for the proinflammatory (M1) phenotype, a decrease in expression during aging cannot simply be explained by a shift toward a proinflammatory phenotype during aging (Lee et al., 2013; Schuitemaker et al., 2012).

An alternative explanation for the observed decrease in expression levels of the microglia module is a lower rate of transcription for genes that regulate microglia activation. It has been observed, for example, in the present and in previous studies (Harry, 2013; Wynne et al., 2010), that expression of receptor *CX*_*3*_*CR1* is diminished in the aged brain. Along these lines morphological changes in microglia during older age (Conde and Streit, 2006; Hefendehl et al., 2014; Streit et al., 2004) potentially reflect dystrophy and cellular senescence rather than increased activation, which could explain a downregulation of endogenous receptor levels including for CX_3_CR1 (Harry, 2013).

The microglia module in adult human brains recapitulates MRs for microglia development during embryogenesis (Kierdorf and Prinz, 2013). This indicates that the identified MRs maintain a causal role for microglia homeostasis during aging. One possible role the MRs could fulfil for microglia in adult brains is regulation of replicative senescence. For example, given PU.1's role in preventing excessive hematopoietic stem cell division (Staber et al., 2013), this known MR for early microglia development may also mediate microglia replicative senescence in normal aging. Accordingly, TAL1 is directly linked to cell growth rates (Dey et al., 2010). In line with this basic notion, microglia senescence during aging (Streit et al., 2008) and expression levels of *PU.1* (Furukawa et al., 2004), *RUNX1* (Adamo et al., 2009), and *TAL1* (Wang et al., 2006) are all affected by stress (Furukawa et al., 2004). Therefore, although *PU.1* sustains its levels via autoregulation (Staber et al., 2013), it could prevent excessive proliferation of M1 microglia in response to accumulating oxidative stress during aging in co-ordination with other MRs.

## Disclosure statement

The authors have no conflicts of interest to disclose.
