# Supplementary material for: Microglia recapitulate a hematopoietic master regulator network in the aging human frontal cortex
Source: Neurobiol Aging. 2015 Aug;36(8):2443.e9–2443.e20. doi: 10.1016/j.neurobiolaging.2015.04.008 (PMC4503803; doi:10.1016/j.neurobiolaging.2015.04.008)
Supplement: Supplementary Tables 1–7 and Supplementary Figures 1–5 [file mmc1.docx]

**Appendix**

**Supplementary Table S1. Genes in the age-dependent gene module in the braincloud data set (*n*=162).**

ABCC4

ADAM28

ADAP2

ADORA3

ADRB2

AFAP1L1

AJ710809

ALDH1A3

ALOX5

ALOX5AP

ALPK1

APBB1IP

ARHGAP30

ARHGDIB

ARPC1B

ATP8B4

AV718565

B3GNT5

BG181918

BIN2

BLNK

BM990598

BMP6

BTK

C17orf60

C1QA

C1QB

C1QC

C2

C21orf96

C3

C3AR1

C7orf58

C9orf167

CARD6

CASP1

CCR1

CD14

CD163

CD300A

CD33

CD37

CD53

CD68

CD74

CD84

CFD

CLEC7A

CMTM7

CTSC

CX3CR1

CXCL16

CYBA

CYBB

CYTH4

DAPP1

DOCK8

DQ680071

EVI2B

FAM184B

FBP1

FCER1G

FCGR2A

FLT1

FOLR2

FPR1

FYB

GAL3ST4

GPR34

GPR65

GYPC

HAVCR2

HCG22

HCK

HCLS1

HCST

HK2

HLA-DMB

HLA-DOA

HLA-DPA1

HLA-DPB1

HLA-DQB2

HMHA1

IGSF6

IL13RA1

IL18

INPP5D

ITGAX

KCNE3

KIAA1274

KLF6

KLHL6

LAIR1

LAPTM5

LAT2

LCP1

LHFPL2

LILRA2

LILRA4

LILRB1

LOC727797

LOC727924

LPAR5

LTC4S

LY75

LY86

MAF

MNDA

MPEG1

MS4A4A

MS4A6A

MS4A7

MYOF

NCF4

NCKAP1L

NLRC5

NLRP1

OLFML3

P2RY13

P2RY5

PCDH12

PGDS

PIK3R5

PLCG2

PLD4

PLEK

PTAFR

PTGS1

PYCARD

RAB32

RASAL3

RBM47

RGS10

RGS18

RGS19

RNASE6

RNASET2

SASH3

SCIN

SELPLG

SLC2A5

SLC7A7

SLCO2B1

SUSD3

TAL1

TBXAS1

TLR1

TLR2

TLR5

TLR7

TLR8

TMEM106A

TMEM156

TMOD3

TNFAIP8L2

TNFRSF1B

TREM2

TRIM38

VAMP8

VPS37C

WAS

WDFY4

**Supplementary Table S2. Genes in the age-dependent gene module in the MRC/UK data set (*n*=141).**

ABI3

ACY3

ADAM28

ADAP2

ADORA3

AIF1

ALOX5

ALOX5AP

APBB1IP

ARHGAP15

B4GALT1

BLNK

BTK

C12orf75

C1QA

C1QB

C1QC

C3

C3AR1

C7orf58

CASP8

CCR1

CD14

CD37

CD4

CD68

CD74

CD84

CD86

CIITA

CLEC7A

CLEC9A

CPVL

CSF1R

CX3CR1

CYBB

CYSLTR1

CYTH4

DHRS9

DOCK2

DOCK8

FAM129A

FCER1G

FCGR1A

FCGR2A

FCGR3A

FOLR2

FYB

GAB3

GPR183

GPR34

GPR65

HAVCR2

HCK

HCLS1

HLA-DMA

HLA-DMB

HLA-DPA1

HLA-DRA

HPGDS

IGSF10

IGSF6

IL10RA

IL18

INPP5D

IRF8

ITGAL

ITGAM

ITGAX

ITGB2

KCTD12

LAIR1

LAPTM5

LCP1

LILRA1

LILRA4

LILRB1

LILRB4

LIPG

LOC100233209

LOC285758

LPAR5

LPAR6

LST1

LY86

MMRN1

MNDA

MPEG1

MS4A14

MS4A4A

MS4A6A

MSR1

MYO1F

NCF4

NCKAP1L

NPY

OLFML3

P2RY12

P2RY13

PARVG

PIK3AP1

PLCB2

PLCG2

PLD4

PLEK

PTGS1

PTPN6

PTPRC

RGS10

RHBDF2

RNASE6

RUNX1

SAMSN1

SELPLG

SIPA1

SLA

SLC2A5

SLC7A7

SLCO2B1

SPI1

STK10

SYK

TAGAP

TBXAS1

TFEC

TGFB1

TLR1

TLR10

TLR5

TLR6

TLR7

TMEM119

TMEM156

TMEM173

TREM2

TYROBP

UBA7

UCP2

VAV1

VSIG4

WDFY4

**Supplementary Table S3. Genes in the age-dependent gene module in the HBTRC data set (*n*=326).**

ABCC4

ACOT9

ACSL5

ADAM28

ADAMTS9

ADH1C

ADORA3

ADRB2

AIF1

ALG9

ALOX5

ALOX5AP

ALPK1

ANXA2

AOAH

APBB1IP

AREG

ARHGAP15

ARHGAP18

ARHGAP30

ARHGDIB

ARPC1B

ATP2B4

ATP8B4

ATXN7L1

B3GNT5

BIN2

BLNK

BTG1

BTK

C10orf10

C1QA

C1QB

C1S

C1orf162

C2

C3

C3AR1

C7

CALCA

CAP1

CASP1

CASP4

CCL20

CCR1

CCR5

CD14

CD163

CD300A

CD300C

CD300LF

CD33

CD37

CD53

CD74

CD84

CD86

CDK2

CDKN1A

CEACAM1

CEBPB

CHEK2

CHODL

CISH

CLEC2B

CLEC4A

CLEC7A

CLEC9A

CLIC1

CMKLR1

CMTM2

CMTM7

COL8A2

COL9A1

CP

CPVL

CRISPLD2

CSF1R

CSF2RA

CSF3R

CSNK1D

CSTA

CTSC

CTSS

CX3CR1

CXCL16

CXCL5

CYBA

CYBB

CYFIP1

CYP2S1

CYTL1

DAPP1

DEF6

DENND3

DOCK11

DOCK2

DOCK8

DSC2

EBI3

ELF4

EMB

EMP1

ENPEP

EVI2B

FABP4

FAM19A5

FBP1

FCER1G

FCGBP

FCGR2A

FCGR2C

FCGR3A

FCGR3B

FGD2

FOLR2

FPR1

FYB

GADD45B

GBP2

GIT2

GMFG

GMIP

GPR4

GPR65

GPSM3

GRAP2

HAMP

HAVCR2

HCK

HCLS1

HIST1H2AI

HIST1H2AM

HIST1H2BG

HLA-DMA

HLA-DMB

HLA-DOA

HLA-DPA1

HLA-DQA1

HLA-DQB1

HLA-DRA

HLA-DRB1

HLA-DRB2

HLA-DRB3

HLA-DRB4

HLA-DRB6

HMHA1

HMOX1

HPSE

IFI30

IFITM2

IFITM3

IGSF6

IL10RA

IL13RA1

IL15RA

IL16

IL18

IL1B

IL4I1

IL4R

IL6

IRAK3

IRF5

IRF8

ITGAM

ITGB2

KCNK13

KCTD12

KIAA0247

KLHL6

KYNU

LAIR1

LAPTM5

LAT2

LCP1

LCP2

LGALS9

LHFPL2

LILRA1

LILRA2

LILRB2

LILRB3

LILRB4

LIMK2

LIPG

LPL

LST1

LY75

LY86

LY96

MAF

MANBA

MCF2L2

MELK

MMP8

MNDA

MR1

MS4A4A

MS4A6A

MS4A7

MSR1

MT1A

MTHFD2

MVP

MYC

MYD88

MYH1

MYO1F

MYO1G

NAPSB

NCF2

NCF4

NCKAP1L

NMI

NUBP1

OLFML3

OSCAR

OSM

P2RY13

P2RY6

PARVG

PDC

PDPN

PECAM1

PI15

PIK3AP1

PIK3CG

PIM1

PLA1A

PLA2G4A

PLAC8

PLAUR

PLB1

PLCG2

PLEK

PLEK2

PRAM1

PROM1

PROS1

PSTPIP2

PTGS1

PTPN6

PTPN7

PTPRC

PYGL

RAB20

RAB25

RAB27A

RAC2

RGS10

RGS16

RGS18

RIPK3

RNASE2

RNASE3

RNASE6

RNASET2

RPGRIP1

RPL23

RPS6KA1

RUNX1

S100A9

S100Z

SAMSN1

SCIN

SECTM1

SERPINA1

SERPINA3

SERPINA5

SERPING1

SFN

SIGLEC10

SIGLEC7

SLA

SLC11A1

SLC1A5

SLC25A19

SLC2A5

SLC37A2

SLC7A7

SOAT1

SOD2

SOX4

SQRDL

ST14

ST6GAL1

STAT3

STC1

STX11

SULT1B1

SUSD3

SYK

TAGAP

TBXAS1

TDGF1

TEAD4

TES

TFEC

THBD

THBS1

TIMP1

TKTL1

TLR1

TLR10

TLR2

TLR7

TLR8

TMEM71

TMEM74

TNFRSF1B

TNFSF13B

TNNI2

TREM2

TRIM34

TRPC4

TTN

TUBB6

TXNDC5

TYROBP

VAMP8

VAV1

VSIG4

WASF3

WDFY4

WEE1

Supplementary Table S4. Functional enrichment for GO-terms for genes contributing to the age-dependent module in all three data sets combined (*n*=426 genes), calculated using a hypergeometric test in GREAT [[1](#_ENREF_1)]. P-values were corrected for multiple testing by applying false discovery rate (FDR Q-Val). ‘Fold’ indicates fold enrichment.

| **Term Name** | **Hypergeometric test** | |
| --- | --- | --- |
| **GO Biological Processes** | **FDR Q-Val** | **Fold** |
| immune response | 4.4E-22 | 4.9 |
| defense response | 9.9E-21 | 4.5 |
| immune system process | 4.8E-19 | 3.4 |
| regulation of immune response | 4.7E-16 | 5.4 |
| activation of immune response | 5.7E-16 | 7.8 |
| positive regulation of immune response | 5.6E-16 | 6.8 |
| positive regulation of immune system process | 3.6E-14 | 5.0 |
| regulation of immune system process | 1.8E-13 | 3.8 |
| innate immune response | 7.8E-13 | 5.3 |
| response to wounding | 3.0E-12 | 3.3 |
| response to stress | 3.6E-12 | 2.2 |
| inflammatory response | 1.2E-09 | 5.2 |
| immune response-activating signal transduction | 3.4E-09 | 6.7 |
| adaptive immune response | 5.7E-09 | 9.6 |
| immune response-regulating signaling pathway | 6.8E-09 | 6.4 |
| immune effector process | 7.8E-09 | 6.8 |
| antigen processing and presentation of peptide or polysaccharide antigen via MHC class II | 1.2E-08 | 36.6 |
| regulation of response to stimulus | 1.2E-08 | 2.1 |
| adaptive immune response based on somatic recombination of immune receptors built from immunoglobulin superfamily domains | 2.0E-08 | 9.6 |
| positive regulation of response to stimulus | 2.5E-08 | 2.9 |
| immune response-activating cell surface receptor signaling pathway | 2.8E-07 | 7.9 |
| immune response-regulating cell surface receptor signaling pathway | 6.6E-07 | 7.4 |
| immunoglobulin mediated immune response | 1.8E-06 | 11.0 |
| B cell mediated immunity | 3.2E-06 | 10.3 |
| antigen receptor-mediated signaling pathway | 9.4E-06 | 7.3 |
| humoral immune response | 1.3E-05 | 8.9 |
| lymphocyte mediated immunity | 2.9E-05 | 8.3 |
| positive regulation of cell activation | 4.6E-05 | 4.6 |
| leukocyte mediated immunity | 5.7E-05 | 6.8 |
| regulation of cytokine production | 6.9E-05 | 3.8 |
| positive regulation of leukocyte activation | 1.5E-04 | 4.5 |
| cellular response to cytokine stimulus | 2.7E-04 | 4.0 |
| regulation of cell activation | 3.0E-04 | 3.6 |
| complement activation | 3.1E-04 | 12.6 |
| regulation of interleukin-8 production | 4.3E-04 | 11.9 |
| regulation of leukocyte activation | 5.7E-04 | 3.6 |
| positive regulation of cytokine production | 5.6E-04 | 4.9 |
| positive regulation of lymphocyte activation | 8.5E-04 | 4.4 |
| antigen processing and presentation of peptide antigen via MHC class II | 9.1E-04 | 33.6 |
| regulation of lymphocyte activation | 1.1E-03 | 3.8 |
| positive regulation of T cell activation | 1.8E-03 | 4.7 |
| regulation of body fluid levels | 2.9E-03 | 2.6 |
| protein activation cascade | 3.3E-03 | 8.5 |
| response to cytokine stimulus | 3.7E-03 | 3.1 |
| regulation of defense response | 3.8E-03 | 3.1 |
| wound healing | 3.7E-03 | 2.5 |
| positive regulation of multicellular organismal process | 3.8E-03 | 2.8 |
| cellular response to mechanical stimulus | 3.7E-03 | 8.2 |
| positive regulation of defense response | 3.7E-03 | 4.0 |
| cell activation | 3.7E-03 | 2.6 |
| regulation of T cell activation | 4.4E-03 | 3.9 |
| leukocyte activation | 4.5E-03 | 3.3 |
| complement activation, classical pathway | 4.5E-03 | 13.6 |
| microglial cell activation involved in immune response | 4.6E-03 | 43.1 |
| cytokine-mediated signaling pathway | 5.3E-03 | 3.8 |
| T cell receptor signaling pathway | 5.6E-03 | 6.3 |
| complement activation, alternative pathway | 6.6E-03 | 19.2 |
| regulation of interleukin-6 production | 6.7E-03 | 7.3 |
| humoral immune response mediated by circulating immunoglobulin | 7.5E-03 | 12.0 |
| regulation of response to external stimulus | 9.6E-03 | 3.2 |
| cellular response to molecule of bacterial origin | 9.9E-03 | 6.8 |
| T cell costimulation | 1.0E-02 | 6.7 |
| icosanoid biosynthetic process | 1.0E-02 | 8.3 |
| regulation of innate immune response | 1.0E-02 | 3.8 |
| positive regulation of tumor necrosis factor production | 1.1E-02 | 10.9 |
| prostanoid metabolic process | 1.2E-02 | 10.7 |
| blood coagulation | 1.2E-02 | 2.6 |
| leukotriene production involved in inflammatory response | 1.2E-02 | 30.2 |
| antigen processing and presentation of exogenous peptide antigen via MHC class II | 1.2E-02 | 30.2 |
| negative regulation of granulocyte differentiation | 1.2E-02 | 30.2 |
| regulation of response to stress | 1.3E-02 | 2.3 |
| hemostasis | 1.3E-02 | 2.5 |
| unsaturated fatty acid biosynthetic process | 1.4E-02 | 7.6 |
| positive regulation of innate immune response | 1.5E-02 | 4.2 |
| activation of innate immune response | 1.4E-02 | 4.7 |
| positive regulation of B cell differentiation | 1.5E-02 | 27.5 |
| leukocyte migration | 1.5E-02 | 3.8 |
| B cell receptor signaling pathway | 1.8E-02 | 9.5 |
| positive regulation of interleukin-8 production | 2.4E-02 | 13.0 |
| cellular response to biotic stimulus | 2.4E-02 | 5.6 |
| regulation of actin polymerization or depolymerization | 3.1E-02 | 5.4 |
| microglial cell activation | 3.1E-02 | 21.6 |
| antigen processing and presentation of exogenous peptide antigen | 3.1E-02 | 21.6 |
| positive regulation of interleukin-8 biosynthetic process | 3.1E-02 | 21.6 |
| antigen processing and presentation | 3.1E-02 | 4.7 |
| leukocyte chemotaxis | 3.2E-02 | 6.4 |
| positive regulation of production of molecular mediator of immune response | 3.5E-02 | 11.5 |
| macrophage activation involved in immune response | 3.6E-02 | 20.1 |
| inositol trisphosphate metabolic process | 3.6E-02 | 20.1 |
| regulation of actin filament length | 3.7E-02 | 5.2 |
| cellular response to lipopolysaccharide | 4.0E-02 | 6.1 |
| myeloid cell activation involved in immune response | 4.2E-02 | 10.9 |
| regulation of interferon-beta production | 4.2E-02 | 10.9 |
| regulation of tumor necrosis factor production | 4.3E-02 | 6.0 |
| icosanoid metabolic process | 4.5E-02 | 5.9 |
| leukocyte activation involved in immune response | 4.7E-02 | 5.9 |
| positive regulation of type IIa hypersensitivity | 4.8E-02 | 50.3 |
| detection of triacyl bacterial lipopeptide | 4.8E-02 | 50.3 |
| detection of diacyl bacterial lipopeptide | 4.8E-02 | 50.3 |
| detection of bacterial lipopeptide | 4.8E-02 | 50.3 |
| cellular response to diacyl bacterial lipopeptide | 4.8E-02 | 50.3 |
| cellular response to triacyl bacterial lipopeptide | 4.8E-02 | 50.3 |
| antigen processing and presentation of exogenous antigen | 4.6E-02 | 17.8 |
| **GO Molecular Function** |  |  |
| receptor activity | 2.2E-04 | 2.1 |
| transmembrane signaling receptor activity | 5.4E-03 | 2.3 |
| signaling receptor activity | 2.0E-02 | 2.1 |
| **GO Cellular Components** |  |  |
| MHC class II protein complex | 5.5E-06 | 33.6 |
| complement component C1 complex | 5.4E-04 | 75.5 |
| MHC protein complex | 1.2E-03 | 12.1 |
| lysosomal membrane | 2.9E-02 | 4.7 |
| lysosome | 3.5E-02 | 3.0 |
| Fc-epsilon receptor I complex | 4.4E-02 | 50.3 |
| Toll-like receptor 1-Toll-like receptor 2 protein complex | 4.4E-02 | 50.3 |
| perinuclear endoplasmic reticulum | 4.4E-02 | 50.3 |
| NADPH oxidase complex | 4.4E-02 | 16.8 |

Supplementary Figure S1. Scatterplot of Pearson’s correlation coefficient (y-axis) for the correlation between module eigengene (ME) and age [years] at constant RIN values (x-axis).

**
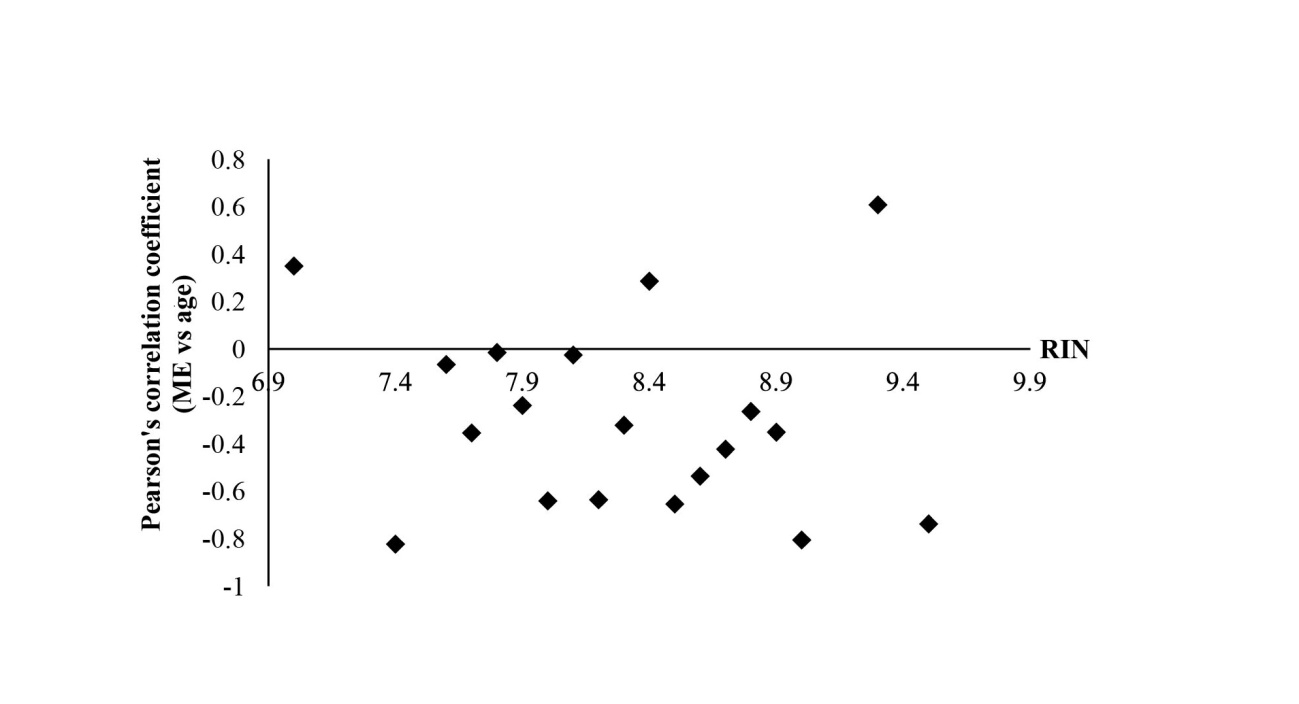
**

**
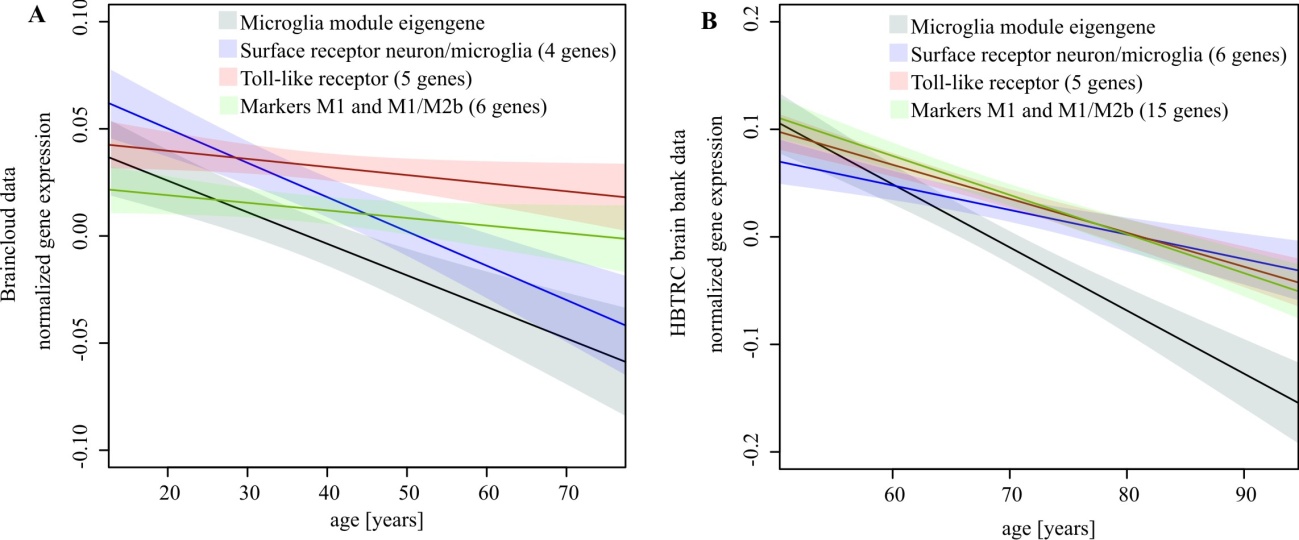
**

Supplementary Figure S2. Microglia surface receptors’ and microglia M1 and M1/M2b phenotype markers’ expression correlation with age for (A) braincloud data and (B) HBTRC brain bank data. The regression model fit and confidence interval for average gene expression versus age are for the microglia module’s eigengene (grey), surface receptors for neuron-microglia crosstalk (blue), Toll-like receptors (red) or M1 and M1/M2b activational phenotype markers (green). The x-axis shows age and the y-axis shows normalized gene expression of the microglia module’s ME.

**Supplementary Table S5. Gene names for marker genes indicating a given cell type or microglia phenotype [**[**2-5**](#_ENREF_2)**].**

| **Gene** | **cell type/phenotype** |
| --- | --- |
| *FCGR2A* | M1 |
| *FCGR2B* | M1 |
| *FCGR3B* | M1 |
| *CXCL10* | M1 |
| *CCL15* | M1 |
| *CCL19* | M1 |
| *CCL8* | M1 |
| *FCGR3A* | M1 |
| *CXCL11* | M1 |
| *CXCL9* | M1 |
| *IL15* | M1 |
| *IL12A* | M1 |
| *IL12B* | M1 |
| *IL1A* | M1 |
| *IL23A* | M1 |
| *NOS2* | M1 |
| *CD74* | M1 and M2b |
| *CD86* | M1 and M2b |
| *HLA-DMA* | M1 and M2b |
| *HLA-DMB* | M1 and M2b |
| *HLA-DPA1* | M1 and M2b |
| *HLA-DPB1* | M1 and M2b |
| *HLA-DQA2* | M1 and M2b |
| *HLA-DRA* | M1 and M2b |
| *HLA-DOB* | M1 and M2b |
| *HLA-DQB1* | M1 and M2b |
| *IL6* | M1 and M2b |
| *HLA-DQB2* | M1 and M2b |
| *HLA-DRB1* | M1 and M2b |
| *HLA-DRB3* | M1 and M2b |
| *HLA-DRB4* | M1 and M2b |
| *HLA-DRB5* | M1 and M2b |
| *TNF* | M1 and M2b |
| *CXCL13* | M1 and M2c |
| *CCL20* | M1 or M2b |
| *IGF1* | M2a |
| *PDGFB* | M2a |
| *PDGFC* | M2a |
| *TGFB1* | M2a |
| *CCL13* | M2a |
| *CCL14* | M2a |
| *CCL17* | M2a |
| *CCL22* | M2a |
| *CCL23* | M2a |
| *CCL24* | M2a |
| *CCL26* | M2a |
| *FN1* | M2a |
| *IL1RN* | M2a |
| *PDGFA* | M2a microglia_oligodendrocyte |
| *RETNLB* | M2a |
| *IL10* | M2a and M2b |
| *MSR1* | M2a and M2c |
| *MRC1* | M2a and M2c |
| *ARG1* | M2a or M2c |
| *CCL18* | M2a or M2c |
| *CD163* | M2a, M2b and M2c |
| *CCL1* | M2b |
| *CXCL2* | M2b |
| *CXCL3* | M2b |
| *SPHK1* | M2b |
| *IL1B* | M2b |
| *CCL16* | M2c |
| *CD68* | microglia |
| *PTPRC* | microglia |
| *P2RY12* | microglia unique |
| *TMEM119* | microglia unique |
| *OLFML3* | microglia unique |
| *P2RY13* | microglia unique |
| *CX3CR1* | microglia unique |
| *GPR34* | microglia unique |
| *HEXB* | microglia unique |
| *RHOB* | microglia unique |
| *JUN* | microglia unique |
| *RAB3IL1* | microglia unique |
| *SERPINE2* | microglia unique |
| *CCL2* | microglia unique |
| *FCRLS* | microglia unique |
| *SCOC* | microglia unique |
| *SIGLECH* | microglia unique |
| *SLC2A5* | microglia unique |
| *LRRC3* | microglia unique |
| *PLXDC2* | microglia unique |
| *USP2* | microglia unique |
| *CTSF* | microglia unique |
| *CTTNBP2NL* | microglia unique |
| *TGFBR1* | microglia unique |
| *ATP8A2* | microglia unique |
| *LGMN* | microglia unique |
| *SLCO2B1* | microglia unique |
| *MAFB* | microglia unique |
| *EGR1* | microglia unique |
| *BHLHE41* | microglia unique |
| *FCGR1A* | microglia unique |
| *HPGDS* | microglia unique |
| *CTSD* | microglia unique |
| *HSPA1A* | microglia unique |
| *LAG3* | microglia unique |
| *CSF1R* | microglia unique |
| *ADAMTS1* | microglia unique |
| *F11R* | microglia unique |
| *GOLM1* | microglia unique |
| *NUAK1* | microglia unique |
| *CRYBB1* | microglia unique |
| *LTC4S* | microglia unique |
| *ASPH* | microglia_neuron |
| *BASP1* | microglia_neuron |
| *CD34* | microglia_neuron |
| *TANC2* | microglia_neuron |
| *SLC7A8* | microglia_neuron |
| *SYNGR1* | microglia_neuron |
| *RGMB* | microglia_neuron |
| *NPNT* | microglia_neuron |
| *MEF2A* | microglia_neuron |
| *RTN4RL1* | microglia_neuron |
| *MEF2C* | microglia_neuron |
| *KHDRBS3* | microglia_neuron |
| *MYO1B* | microglia_neuron |
| *RTN1* | microglia_neuron |
| *SALL1* | microglia_astrocyte |
| *LTGB5* | microglia_astrocyte |
| *CST3* | microglia_astrocyte |
| *SLC1A3* | microglia_astrocyte |
| *GPR56* | microglia_astrocyte |
| *MERTK* | microglia_astrocyte |
| *CABLES1* | microglia_astrocyte |
| *SALL3* | microglia_astrocyte |
| *ETV5* | microglia_astrocyte |
| *CHST7* | microglia_astrocyte |
| *EYA4* | microglia_astrocyte |
| *ARHGAP5* | microglia_astrocyte |
| *RNF180* | microglia_astrocyte |
| *PMP22* | microglia_astrocyte |
| *TMEM144* | microglia_astrocyte |
| *NPL* | microglia_astrocyte |
| *TRIM47* | microglia_astrocyte |
| *TLR3* | microglia_astrocyte |
| *LTGA6* | microglia_astrocyte |
| *ABCA1* | microglia_astrocyte |
| *TMEM100* | microglia_astrocyte |
| *ARHGAP12* | microglia_astrocyte |
| *FADS1* | microglia_astrocyte |
| *SPIRE1* | microglia_astrocyte |
| *TSPAN7* | microglia_astrocyte |
| *JAM2* | microglia_astrocyte |
| *LRRC8A* | microglia_astrocyte |
| *IL18* | microglia_astrocyte |
| *C1QC* | microglia_oligodendrocyte |
| *C1QB* | microglia_oligodendrocyte |
| *C1QA* | microglia_oligodendrocyte |
| *CTSS* | microglia_oligodendrocyte |
| *TIMP2* | microglia_oligodendrocyte |
| *BIN1* | microglia_oligodendrocyte |
| *FRMD4B* | microglia_oligodendrocyte |
| *TMCC3* | microglia_oligodendrocyte |
| *CHN2* | microglia_oligodendrocyte |
| *GAB1* | microglia_oligodendrocyte |
| *SPSB1* | microglia_oligodendrocyte |
| *SLC12A2* | microglia_oligodendrocyte |
| *SCARB2* | microglia_oligodendrocyte |
| *RAP1GDS1* | microglia_oligodendrocyte |
| *PAK1* | microglia_oligodendrocyte |
| *EPN2* | microglia_oligodendrocyte |
| *GLT1* | astrocyte |
| *CX30* | astrocyte |
| *GFAP* | astrocyte |
| *GLAST* | astrocyte |
| *AQP4* | astrocyte |
| *ALDOC* | astrocyte |
| *FGFR3* | astrocyte |
| *NG2* | oligodendrocyte |
| *SOX10* | oligodendrocyte |
| *CX47* | oligodendrocyte |
| *MBP* | oligodendrocyte |
| *MOG* | oligodendrocyte |
| *UGT8A* | oligodendrocyte |
| *GAL3ST1* | oligodendrocyte |
| *MOBP* | oligodendrocyte |
| *MAG* | oligodendrocyte |
| *MAL* | oligodendrocyte |
| *NEFL* | neuron |
| *GABRA1* | neuron |
| *SYT1* | neuron |
| *KCC2* | neuron |
| *SNAP25* | neuron |
| *KCNQ2* | neuron |
| *SV2B* | neuron |
| *OCLN* | endothelial |
| *VWF* | endothelial |
| *TIE2* | endothelial |
| *CLDN5* | endothelial |
| *PECAM1* | endothelial |

**Supplementary Table S6. Transcription factors identified as master regulators for the microglia module when results for all three data sets were combined.**

| **Master regulators** |
| --- |
| *ARNTL2* |
| *ATF4* |
| *BACH1* |
| *BATF* |
| *BATF3* |
| *BCL3* |
| *BCL6* |
| *BNC2* |
| *BPNT1* |
| *CASZ1* |
| *CBFB* |
| *CEBPB* |
| *CEBPD* |
| *CREB3L2* |
| *DACH2* |
| *DLX6* |
| *DOT1L* |
| *DRGX* |
| *DUSP12* |
| *E2F6* |
| *ELF4* |
| *ELK3* |
| *FLI1* |
| *FOS* |
| *FOXC1* |
| *HEYL* |
| *HHEX* |
| *HIF1A* |
| *HLA-DQB2* |
| *HLX* |
| *HOXD4* |
| *IFI16* |
| *IKZF1* |
| *INF2* |
| *IRF1* |
| *IRF5* |
| *IRF8* |
| *JUNB* |
| *KLF12* |
| *KLF6* |
| *LYL1* |
| *MAF* |
| *MAFB* |
| *MAFF* |
| *MYC* |
| *NEUROD6* |
| *NEUROG2* |
| *NFATC2* |
| *NFATC3* |
| *NFE2* |
| *NFIL3* |
| *NFKBIA* |
| *NFKBIZ* |
| *NONO* |
| *NR3C2* |
| *PARP12* |
| *PLEK* |
| *PRDM16* |
| *PRDM4* |
| *RNASE2* |
| *RNF113A* |
| *RREB1* |
| *RUNX1* |
| *RUNX3* |
| *SATB2* |
| *SF3A2* |
| *SIX4* |
| *SLC29A3* |
| *SLC4A10* |
| *SOX11* |
| *SOX15* |
| *SOX4* |
| *SP100* |
| *SPI1 (PU.1)* |
| *STAT3* |
| *STAT4* |
| *STAT5A* |
| *STAT6* |
| *TAL1* |
| *TBX15* |
| *TBX3* |
| *TEAD4* |
| *TFDP3* |
| *TIAL1* |
| *U2AF1L4* |
| *WDHD1* |
| *XBP1* |
| *ZC3HAV1* |
| *ZFP36* |
| *ZFP36L1* |
| *ZFP36L2* |
| *ZFPM2* |
| *ZIC1* |
| *ZNF14* |
| *ZNF189* |
| *ZNF248* |
| *ZNF32* |
| *ZNF366* |
| *ZNF425* |
| *ZNF521* |
| *ZNF526* |
| *ZNF628* |
| *ZNF687* |
| *ZNF696* |
| *ZNF776* |
| *ZNF821* |
| *ZSCAN16* |

**Supplementary Table S7. Master regulators in the protein-protein-interaction network.**

| ATF4 |
| --- |
| BACH1 |
| BATF |
| BATF3 |
| BCL3 |
| BCL6 |
| CBFB |
| CEBPB |
| ELK3 |
| FOS |
| HIF1A |
| HOXD4 |
| IFI16 |
| IKZF1 |
| IRF1 |
| IRF8 |
| JUNB |
| LYL1 |
| MAF |
| MAFB |
| NFATC2 |
| NFATC3 |
| NFE2 |
| NFIL3 |
| NFKBIZ |
| NR3C2 |
| PARP12 |
| PRDM4 |
| RREB1 |
| RUNX1 |
| RUNX3 |
| SPI1 |
| STAT3 |
| STAT4 |
| STAT5A |
| STAT6 |
| TAL1 |
| XBP1 |
| ZFPM2 |
| ZNF687 |


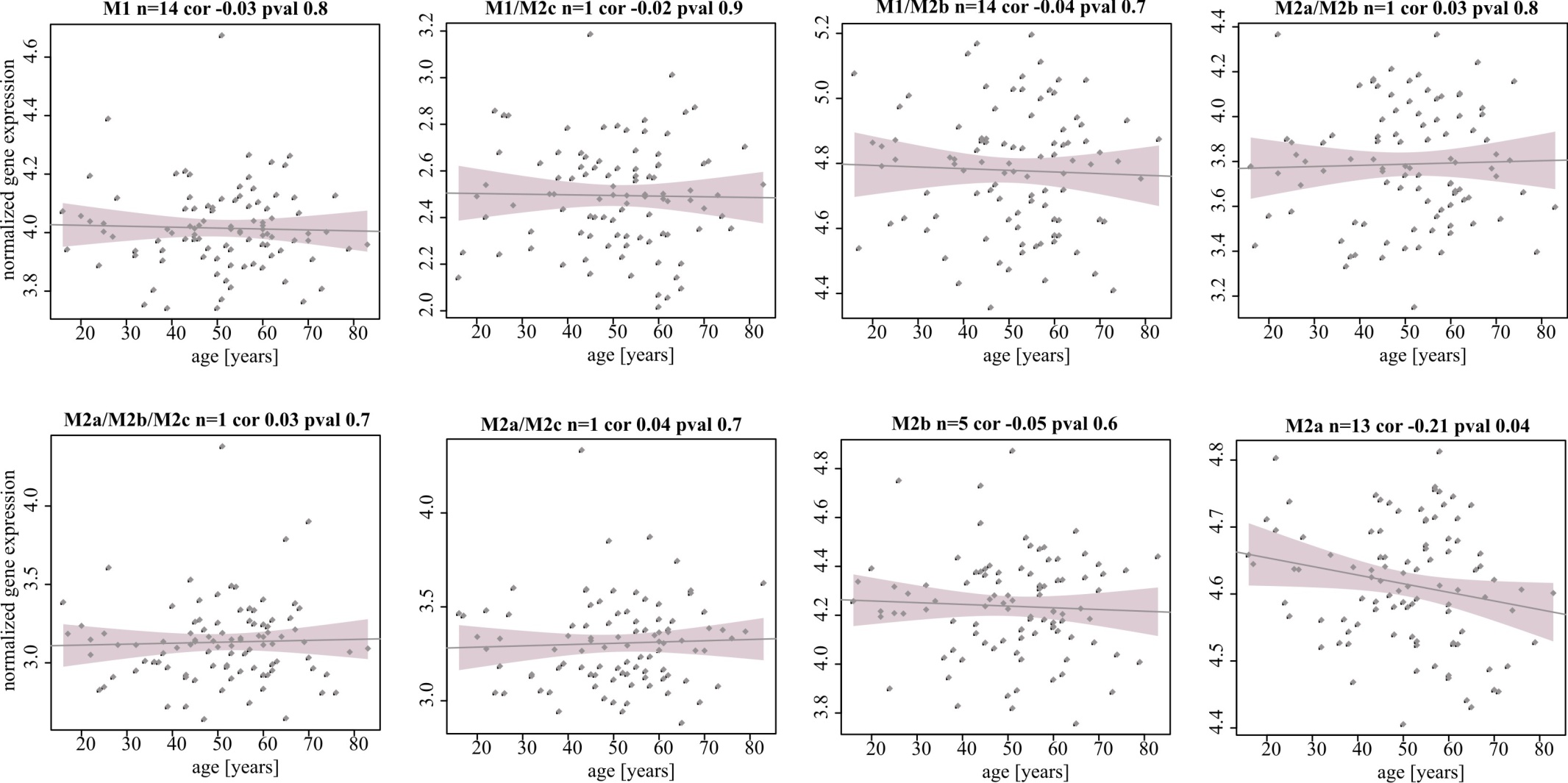


**Supplementary Figure S3. Pearson’s correlation (indicated as “cor” and p-value as “pval”) of age with average expression of all marker genes (number of marker genes indicated as “n”) for specific microglia activation phenotypes (indicated in plots’ titles) in the MRC/UK data.**

**
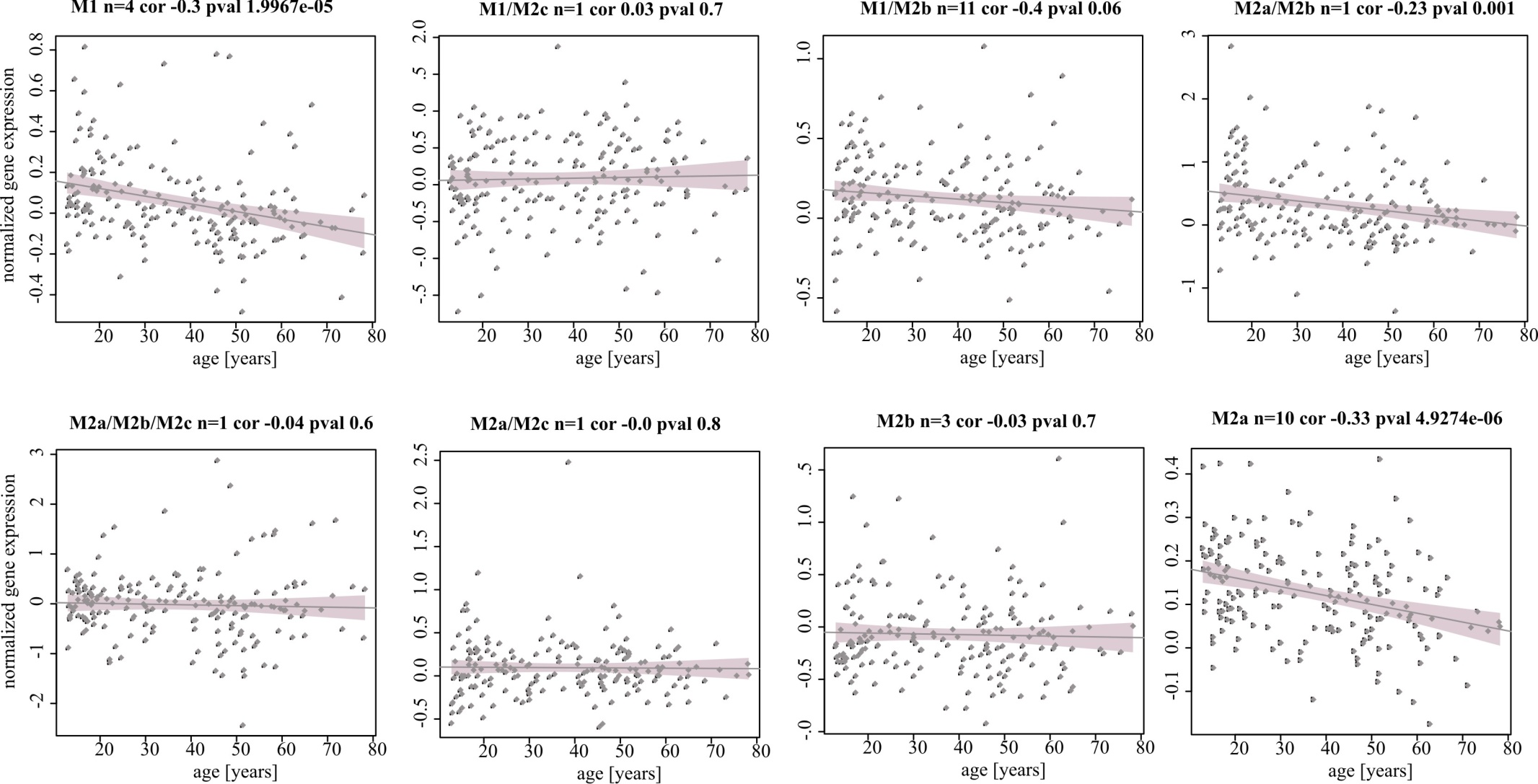
 Supplementary Figure S4. Pearson’s correlation (indicated as “cor” and p-value as “pval”)) of age with average expression of all marker genes (number of marker genes indicated as “n”) for specific microglia activation phenotypes (indicated in plots’ titles) in the braincloud data.**

**
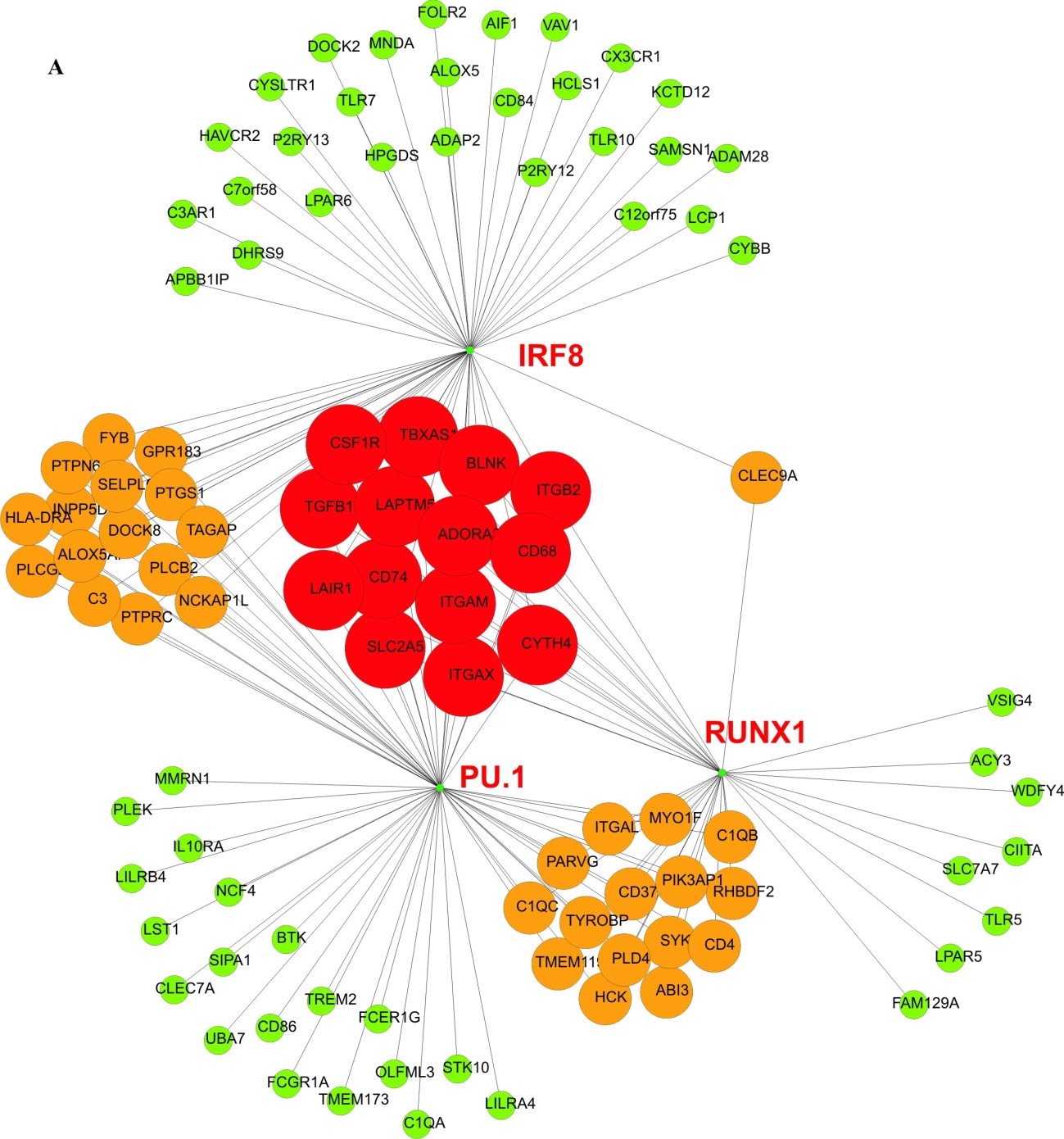
**

**
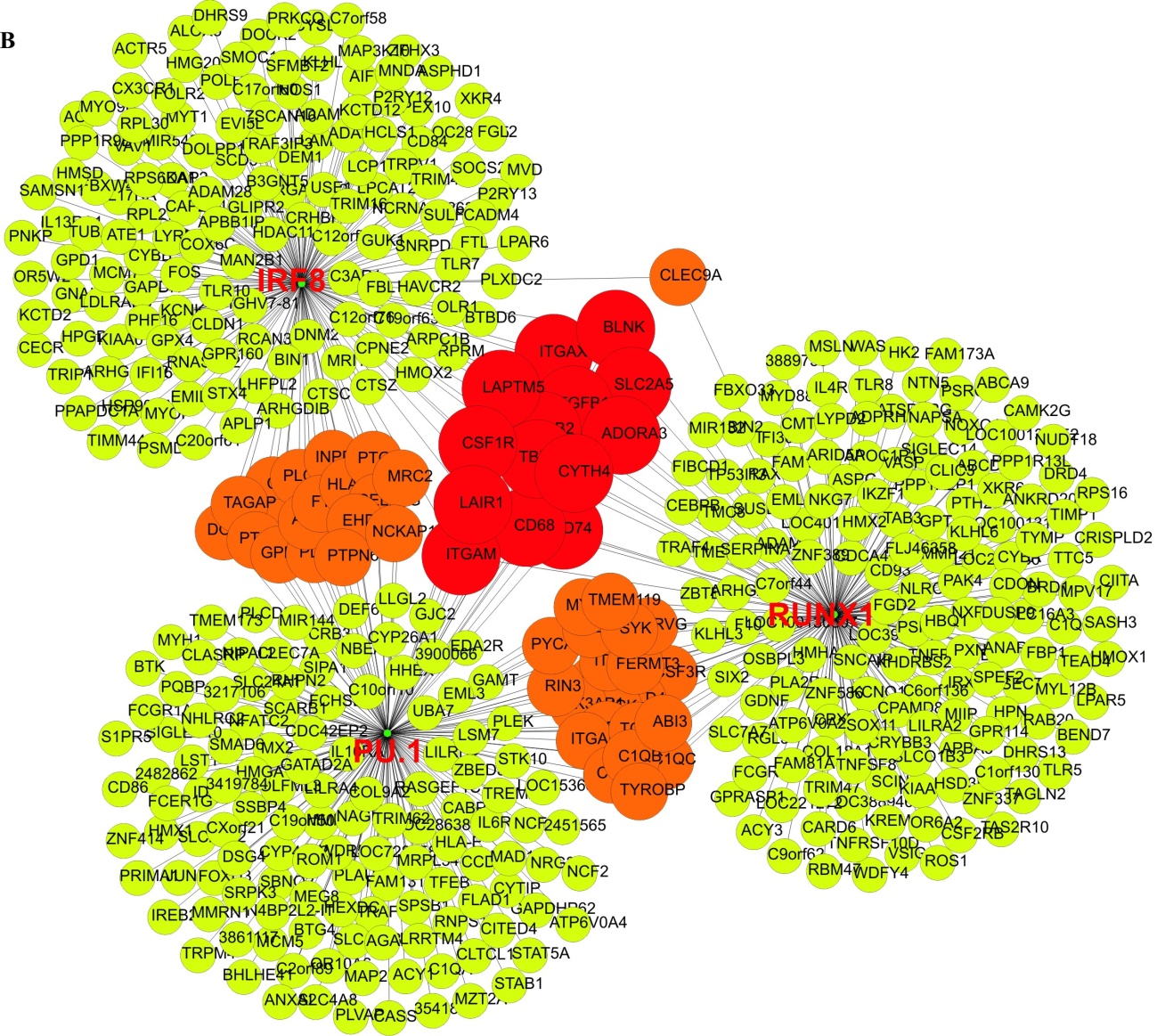
**

Supplementary Figure S5. Overlap of master regulator’s (*RUNX1*, *PU.1* and *IRF8*) predicted target genes only in the microglia module (A) compared to genes from whole network from the MRC/UK data set (B). A) Predicted regulons (in other words target genes) of *RUNX1, PU.1,* and *IRF8* in the MRC/UK gene expression data for predicted regulons which are part of the microglia module. Size and colour of nodes reflect indegree of nodes. More specifically, small and green nodes have a small number of incoming edges, and, by contrast, large and red nodes have a high number of incoming edges. Yellow nodes are intermediate. Here, the possible range of indegree goes from zero (a master regulator which is not regulated by another master regulator and therefore has no incoming edges) to two (an incoming edge from both other master regulators.) For simplicity, only edges that connect at least one of the three master regulators (*RUNX1*, *PU.1* and *IRF8*) with another gene are displayed, excluding edges between target genes. B) Same measures including all predicted target genes across the whole network. It is of note that *n*=14 genes that are regulated by all three master regulators are present in both A and B.
